# Anthocyanins and phenolic compounds in colored wheat grain

**DOI:** 10.18699/vjgb-25-42

**Published:** 2025-06

**Authors:** E.V. Chumanova, T.T. Efremova, K.V. Sobolev, E.A. Kosyaeva

**Affiliations:** Institute of Cytology and Genetics of the Siberian Branch of the Russian Academy of Sciences, Novosibirsk, Russia; Institute of Cytology and Genetics of the Siberian Branch of the Russian Academy of Sciences, Novosibirsk, Russia; Institute of Cytology and Genetics of the Siberian Branch of the Russian Academy of Sciences, Novosibirsk, Russia Novosibirsk State Agrarian University, Novosibirsk, Russia; Institute of Cytology and Genetics of the Siberian Branch of the Russian Academy of Sciences, Novosibirsk, Russia Novosibirsk State Agrarian University, Novosibirsk, Russia

**Keywords:** wheat, blue, purple, black grain, anthocyanins, phenolic compounds, antioxidant activity, пшеница, голубая, фиолетовая, черная окраска зерна, антоцианы, фенольные соединения, антиоксидантная активность

## Abstract

Wheat is an extremely important and preferred source of human nutrition in many regions of the world. The production of biofortified colored-grain wheat varieties, which are known to contain a range of biologically active compounds, including anthocyanins, phenolic compounds, vitamins and minerals, reflects a worldwide trend toward increasing dietary diversity and improving diet quality through the development and introduction of diverse functional foods. The present work describes the genetic systems that regulate the biosynthesis and accumulation of anthocyanins in the pericarp and aleurone layer, the presence of which imparts purple, blue and black grain color. The review is devoted to the systematization of available information on the peculiarities of qualitative and quantitative content of anthocyanins, soluble and insoluble phenolic acids in wheat grain of different color, as well as on indicators of antioxidant activity of alcoholic extracts of grain depending on the content of anthocyanins and phenolic compounds. A huge number of studies have confirmed that these compounds are antioxidants, have anti-inflammatory activity and their consumption makes an important contribution to the prevention of a number of socially significant human diseases. Consumption of colored cereal grain products may contribute to an additional enrichment of bioactive compounds in human diet along with the usual sources of antioxidants. Special attention in the review is paid to the description of achievements of Russia’s breeders in developing promising varieties and lines with colored grain, which will be a key factor in expanding the opportunities of the domestic and international grain market.

## Abstract (ru)

Пшеница является чрезвычайно важным и предпочтительным источником питания человека во многих регионах земного шара. Получение биофортифицированных сортов мягкой пшеницы с окрашенным зерном, которое, как известно, содержит целый ряд биологически активных соединений, в том числе антоцианы, фенольные соединения, витамины и минералы, отражает общемировую тенденцию на увеличение разнообразия и повышение качества рациона путем разработки и внедрения разнообразных продуктов функционального питания. В настоящей работе описаны генетические системы, регулирующие биосинтез и накопление антоцианов в перикарпе и алейроновом слое, присутствие которых придает зерну фиолетовую, голубую и черную окраску. Обзор посвящен систематизации информации об особенностях качественного и количественного содержания антоцианов, растворимых и нерастворимых фенольных кислот в зерне пшеницы с различной окраской, а также показателях антиоксидантной активности спиртовых экстрактов зерна в зависимости от содержания антоцианов и фенольных соединений. Огромным количеством исследований подтверждено, что данные соединения являются антиоксидантами и соединениями с противовоспалительной активностью и их употребление вносит важный вклад в профилактику ряда социально значимых заболеваний человека. Употребление продуктов из окрашенного зерна злаков может способствовать дополнительному обогащению рациона людей биологически активными соединениями наряду с привычными источниками антиоксидантов. Отдельное внимание в обзоре уделено описанию достижений отечественных селекционеров, усилия которых в этой области позволили получить ряд перспективных сортов и линий с окрашенным зерном, которые могут послужить основой создания рынка биофортифицированных диетических продуктов питания в России и увеличения экспортного потенциала рынка зерна.

## Introduction

Wheat occupies an important place in the structure of world consumption. Over the last two decades, the biofortification associated with increasing the nutritional value of food products from wheat grain has become an actual trend in breeding, in particular, much attention of researchers and breeders is focused on obtaining colored-grain wheat varieties, rich in anthocyanins. Depending on the type and accumulation of anthocyanins in different layers of the grain, wheat grain can have purple (in the pericarp, controlled by Pp genes), blue (in the aleurone layer, Ba genes) and dark purple (black) color (in both layers at the same time, Ba + Pp genes).

The value of colored wheat is a more diverse composition of flavonoids with important biological properties (Wang et al., 2020; Razgonova et al., 2021). In addition, many researchers have shown that colored-grain wheat has a higher content of protein and essential amino acids (Tian et al., 2018; Garg et al., 2022), a number of macro- and microelements: Zn, Fe, Mg, K, Ca, Se, Cu an Mn (Ficco et al., 2014; Sharma S. et al., 2018; Tian et al., 2018; Dhua et al., 2021; Shamanin et al., 2024), vitamins B1, B2, B9 and E (Granda et al., 2018) compared to red and white grains. Anthocyanins and phenolic compounds have great antioxidant potential, protecting cells from free radical damage. As well as these compounds have anti-inflammatory and antibacterial activity, preventing the development of diabetes, cardiovascular, neurodegenerative diseases and cancer (Laddomada et al., 2017; Francavilla, Joye, 2020; Mohammadi et al., 2024).

Colored wheat in China, India, Singapore, Canada and Austria is used to produce functional food products from whole wheat flour containing high amounts of antioxidants: different types of whole wheat breads, bakery products, cookies, pasta, pancakes, crackers (Garg et al., 2022; Gamel et al., 2023). However, the content of anthocyanins and phenolic compounds decreases when exposed to high temperatures. According to the literature data, the loss of anthocyanins during bread baking varies between 10–73 % and during the preparation of noodles, pasta, tortillas, biscuits, the content of anthocyanins and phenolic compounds decreases by 29–74 and 26–80 %, respectively (Garg et al., 2022). It has been shown that bakery products from colored grains are not inferior or are even superior to products from uncolored flour in terms of baking and organoleptic properties, and their shelf life increases (Khlestkina et al., 2017).

In recent years in our country, the direction towards the production of anthocyanin-biofortified wheat has been actively developed (Khlestkina et al., 2017; Vasilova et al., 2021; Rubets et al., 2022; Gordeeva et al., 2022; Shamanin et al., 2022, 2024), which forms the idea of a healthy lifestyle and nutrition, since wheat is an important food crop for Russia.

This review compiles available information on genetic factors regulating the accumulation of anthocyanins in colored wheat grain, peculiarities of anthocyanins and phenolic acids content and antioxidant activity (AOA) in colored grain, and summarizes information on the achievements of Russian scientists in obtaining promising colored-grain wheat lines and varieties.

## Genetic control of the synthesis and accumulation of anthocyanins in wheat grain

**Fig. 1.**
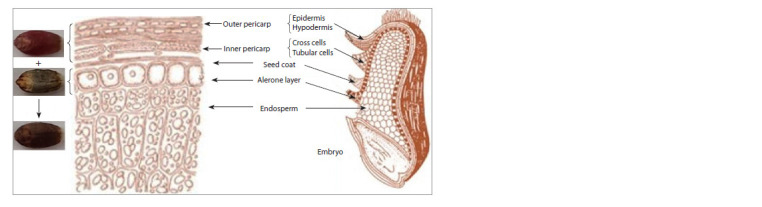
Internal structure of wheat grain: longitudinal and transverse section. Adapted from (Laddomada et al., 2015).

The wheat grain consists of an embryo and endosperm densely surrounded by epidermis and a seed coat (Fig. 1). The fruit sheath (pericarp or pericarpium: etymologically derived from two Greek words, i. e., peri: around and carpos: fruit), consisting of several layers: epidermis, hypodermis, remnants of thin-walled cells, intermediate, transverse and tubular cells, surrounds the grain and plays a protective role. Seed coat cells (pigment layer, testa) of red-grain wheat contain proanthocyanidins that increase grain resistance to preharvest germination (Himi et al., 2011). The aleurone layer of the grain is the outer layer of the endosperm, consisting of a single layer of cells, square or slightly oblong in shape. It derives its name from the content of aleurone grains, which are protein storage structures.

Colored wheat is known to exist in three different forms: blue, purple and dark purple (black), depending upon the types and position of the anthocyanins in kernel layers. The bluishgray color of wheat is because of the synthesis of anthocyanins in the aleurone layer. The presence of purple color, in turn, is due to the accumulation of anthocyanins in pericarp cells. The black grain results from the accumulation of anthocyanins simultaneously in the pericarp and aleurone layer (Fig. 1).

Anthocyanin biosynthesis in the aleurone layer is under the control of dominant alleles of Ba genes (Blue aleurone) localized in chromosomes of the fourth homeologous group of some cereal species. Ba1 (syn. Ba(b)) is localized in the long arm of chromosome 4E (formerly 4Ag) of Thinopyrum ponticum (Podp.) Barkworth & D.R. Dewey (Agropyron elongatum L.; Lophopyrum ponticum (Podb.) Love; Elytrigia pontica (Podp.) Holub) (Zheng et al., 2006). Ba2 (syn. Ba(a)) is localized in the long arm of chromosome 4Abo Triticum boeoticum Boiss. or 4Am T. monococcum L. (Singh et al., 2007). BaThb (syn. Ba(c)) is localized in the chromosome 4J Th. bessarabicum (Sặvul. & Rayss) Á. Lőve (Shen et al., 2013).

ThMyc4E, encoding a MYC-type transcription factor with a bHLH domain is a Ba1 candidate gene (Li N. et al., 2017). TbMyC4A, encoding a bHLH transcription factor containing three regulatory domains (bHLH-MYC_N, HLH and ACT-like) (Liu X. et al., 2021) is considered as a likely Ba(a) candidate gene. It is suggested that the BaThb and Ba1 genes may have a common origin (Burešová et al., 2015), as Th. bessarabicum is a probable donor species of the Eb genome of most polyploid wheatgrass species, including Th. ponticum.

Ba genes were introgressed into the common wheat genome by producing substitution, addition and translocation lines. V.S. Arbuzova et al. (2012) and E.I. Gordeeva et al. (2022) obtained substitution lines of spring bread wheat Saratovskaya 29 (S29) with the replacement of chromosome 4B or 4D with chromosome 4E Th. ponticum. Liu Xin et al. (2020) obtained six substitution lines 4Abo(4B) with the Ba2 gene: Z18-1150, Z18-1195, Z18-1223, Z18-1244, Z18-1289, and Z18-3816. The chromosomal composition of several wheat lines with blue grain was described using in situ hybridization (Burešová et al., 2015). The results showed that six different types of Th. ponticum chromatin introgressions were detected: ditelosomic additions (Blue Norco), ditelosomic substitution (Blue Baart), T4BS.4AgL (UC66049), and different translocations of the distal parts of chromosomal arms of Th. ponticum (Sebesta Blue 1-3). Y. Shen et al. (2013) obtained 157 lines derived from the cross between T. aestivum cv. Chinese Spring and a T. aestivum-Th. bessarabicum amphiploid: they isolated monosomic and disomic addition lines with chromosomes 4J, 4JL, and 4JS, as well as T4DS.4DL-4JL carrying a fragment of chromosome 4J.

A significant problem in blue wheat lines selection is caused by the negative inﬂuence of wheatgrass genes linked to blue aleurone genes (Garg et al., 2016). Therefore, it is preferable to involve in breeding purple-grain donors, which are devoid of such a disadvantage.

The purple color of wheat is because of anthocyanin synthesis in the pericarp layer. The first samples of tetraploid wheat T. aethiopicum Jakubz. (T. turgidum L. subsp. abyssinicum Vavilov) with purple-grain genes were collected by Wittmack in Abyssinia (Northern Ethiopia) in the early 1870s and brought to Europe, from where they further spread to different countries (Eticha et al., 2011). It is noteworthy that landraces of purple wheat are still cultivated in Ethiopia.

Anthocyanin synthesis in pericarp is controlled by the complementary interaction of Pp genes (Purple pericarp): Pp-1 (TaPpm1) and Pp3 (TaPpb1/TaMyc1), which encode different types of transcription factors that activate transcription of structural anthocyanin biosynthesis genes (Jiang W. et al., 2018). Pp-1 is a MYB-like transcription factor with an R2R3 regulatory domain. A set of Pp-1 homoeologous genes in chromosomes of the seventh homeologous group is currently known: Pp-A1 in 7AS (T. aestivum) (Gordeeva et al., 2015), Pp-B1 in 7BS (7B in T. durum, 7S in Aegilops speltoides Tausch.) (Khlestkina et al., 2010), and Pp-D1 in 7DS (T. aestivum) (Tereshchenko et al., 2012).

The dominant allele of the Pp3 gene, localized in the centromeric region of chromosome 2A, encodes a transcription factor with a bHLH regulatory domain (Shoeva et al., 2014). Tissue-specific transcriptional activity of the dominant TaMyc1 allele, which is a likely candidate for Pp3, was shown in the colored pericarp of grains with lower expression levels in coleoptile, scales, and leaves. At the same time, Pp-1 is expressed in many plant tissues (Shoeva et al., 2014; Jiang W. et al., 2018). It was found that TaMyc1 has at least four copies in common wheat. In addition to TaMyc1, three copies, TaMyc2–4 are localized in 2AL, 2BL and 2DL, respectively; however, none of these extra copies are transcribed in the pericarp. Comparison of TaMyc1 expression in near-isogenic lines carrying different combinations of dominant and recessive alleles of Pp-1 and Pp3 showed that the dominant allele Pp-D1 partially suppressed the transcription of TaMyc1 in the pericarp (Shoeva et al., 2014).

Four allelic variants were found in the TaPpm1 coding region: TaPpm1a (dominant, in purple wheat) and TaPpm1b, TaPpm1c, and TaPpm1d, which are nonfunctional due to differently sized insertions that cause frameshift or premature transcription termination (in uncolored wheat). There were six 261-bp tandem repeats in the promoter region of TaPpb1 in the purple-grained varieties (TaPpb1a allele), while there was only one repeat unit present in the uncolored wheat varieties (TaPpb1b) (Jiang W. et al., 2018).

The expression of structural genes involved in the anthocyanin biosynthesis pathway is regulated by the MBW complex, which includes the R2R3-MYB, bHLH, and WD40 proteins. The allelic variations of TaPpm1 influence anthocyanin pigmentation by altering the binding ability with bHLH, whereas variations in the TaPpb1 promoter alter its expression level (Jiang W. et al., 2018).

## Anthocyanins content in colored wheat grain

**Fig. 2.**
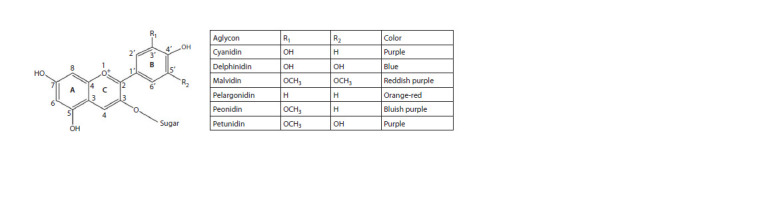
Structure of major anthocyanidins found in wheat grain.

Anthocyanins are water-soluble pigments related to flavonoids that give color to various parts of plants. The basic structure of anthocyanins is shown in Figure 2. Anthocyanidin (aglycone) is the base of the anthocyanin molecule. For most anthocyanins, the sugar moieties, most often glucose, galactose, arabinose and rutinose, are usually connected to anthocyanidins through O-glycosidic bonds at C3 position, sometimes at C3 and C5 positions. In addition, sugars can be acylated by aliphatic and aromatic acids (Francavilla, Joye, 2020). Cyanidin, delphinidin, malvidin, pelargonidin, peonidin, and petunidin are well-known anthocyanidins, which differ from each other in the number of hydroxyl or methoxyl groups (Fig. 2).

The total anthocyanin content (TAC) of wheat widely varies from 10 to 305 μg/g in purple grain, from 17 to 211 μg/g in blue grain and from 56 to 198 μg/g in black grain, but in general, black-grain wheat has a higher anthocyanin content. White and red grain wheat genotypes have the lowest TAC (7–10 μg/g) (Abdel-Aal, Hucl, 2003; Abdel-Aal et al., 2006; Varga et al., 2013; Garg et al., 2016; Kumari et al., 2020; Wang et al., 2020; Iannucci et al., 2022). In addition, whole wheat flour has a lower anthocyanin content compared to the bran fraction (Siebenhandl et al., 2007; Iannucci et al., 2022) (Supplementary Materials, Table S1)1. The anthocyanin content of blue wheat, on average, is higher than that of purple wheat, but some purple wheat contains much more anthocyanins than blue wheat (Abdel-Aal et al., 2016). It has been suggested that in blue grain and black grain, the location of the pigment in deeper layers leads to increased stability of anthocyanins (Garg et al., 2016). In addition, anthocyanins located in the aleurone layer are less firmly bound to cellular components than those in the pericarp, allowing them to be more easily extracted.


Supplementary Materials are available in the online version of the paper: https://vavilov.elpub.ru/jour/manager/files/Suppl_Chuman_Engl_29_3.pdf


It was also found that the qualitative composition of anthocyanins differs between blue- grain and purple-grain wheat. In addition, the main anthocyanins in the genotypes with purple and blue grains are different. It is considered that purple-grain wheat varieties have a more complex anthocyanin composition than blue-grain wheat varieties but a lower TAC. Cyanidin- 3-glucoside is the dominant anthocyanin in purple wheat grain. The most abundant anthocyanins in purple wheat along with cyanidin-3-glucoside are cyanidin-3-galactoside, cyanidin- 3-rutinoside, cyanidin-3-(6ʺ-malonyl glucoside), as well as delphinidin-3-galactoside, malvidin-3-glucoside, peonidin- 3-glucoside, petunidin-3-glucoside, peonidin-3-(6ʺ-malonyl glucoside) (Hosseinian et al., 2008; Abdel-Aal et al., 2018; Jiang Y. et al., 2024; Shamanin et al., 2024).

E.S.M. Abdel-Aal et al. (2006) found eight anthocyanins in purple grain such as cyanidin-3-glucoside, cyanidin-3,5- di-glucoside, peonidin-3-glucoside, and malonyl and succinyl derivatives of cyanidin and peonidin. E.S.M. Abdel-Aal et al. (2018) found a number of other anthocyanins in purple grain: delphinidin-3-rutinoside, malvidin-3-rutinoside, malvidin succinylglucoside, pelargonidin-3-(6ʺ-malonylglucoside), peonidin-3-rutinoside, petunidin-3-(6ʺ-malonylglucoside). F.S. Hosseinian et al. (2008) found the presence of 13 anthocyanins, which included arabinoside derivatives of cyanidin, delphinidin, pelargonidin and peonidin, glucoside derivatives of malvidin, pelargonidin and petunidin, and delphinidin- 3-galactoside.

P. Bartl et al. (2015) identified a number of cyanidin glycosides with a hexose acetylated with malonic and/or acetic acid, delphinidin with a hexose acetylated with coumaric acid, peonidin with a hexose/rhamnose acetylated with malonic and/ or acetic acid, and petunidin with two or three sugar moieties (hexose and rhamnose) acetylated with caffeic or coumaric acid. Y. Jiang et al. (2024) found the presence of 26 anthocyanin glycosides, including 12 acylated ones (acetyl-, malonyl-, and succinyl- derivatives). It was also shown that the TAC and content of individual anthocyanin glycosides increased with size reduction of the flour particle (coarse, fine and superfine flour samples).

The main anthocyanins in blue grains are delphinidin- 3-rutinoside, delphinidin-3-glucoside, malvidin-3-glucoside (Ficco et al., 2014), delphinidin-3-rutinoside, cyanidin-3- glucoside, cyanidin-3-rutinoside (Abdel-Aal et al, 2006), malvinidin- 3-glucoside, delphinidin-3-galactoside, cyanidin- 3-glucoside (Sharma N. et al., 2020), delphinidin-3-rutinoside, delphinidin- 3-glucoside, petunidin-3-glucoside (Iannucci et al., 2022). Blue grain has a high concentration of delphinidin- 3-glucoside (9.9–56.5 μg/g) and delphinidin-3-rutinoside (35.9–72.5 μg/g) (Abdel-Aal et al., 2006; Ficco et al., 2014; Iannucci et al., 2022) or delphinidin-3-galactoside and malvidin- 3-glucoside (Sharma N. et al., 2020). E.S.M. Abdel-Aal et al. (2006) found the presence of eight anthocyanins in bluegrain wheat: cyanidin-3-glucoside, cyanidin-3-rutinoside, delphinidin-3-glucoside, delphinidin-3-rutinoside, malvidin- 3-rutinoside, peonidin-3-rutinoside, petunidin-3-glucoside, and petunidin-3-rutinoside. D.B.M. Ficco et al. (2014) showed the presence of eight anthocyanins and identified new peonidine derivatives. P. Bartl et al. (2015) identified cyanidin, delphinidin, malvidin, peonidin and petunidin derivatives with 1, 2 or 3 hexose moieties with rhamnose or coumaric acid.

Black-grain wheat has not only a higher total anthocyanin content, but also a more diverse composition of anthocyanin glycosides. For example, N. Sharma et al. (2020) found 10 different anthocyanins in blue grain, 6 in purple grain and 11 in black grain. M. Garg et al. (2016) identified 22 different anthocyanins in blue wheat, 23 in purple wheat and 26 in black wheat including cyanidin-3-(6ʺ-succinylglucoside), cyanidin-3-(2Gxylosylrutinoside), cyanidin-3-(3ʺ,6ʺ-dimalonylglucoside), cyanidin-3-(6ʺ-feruloylglucoside)-5-glucoside, cyanidin- 3-rutinoside-3’-glucoside, delphinidin-3-caffeoylglucoside, delphinidin-3-sambubioside, malvidin-3-rutinoside-5-glucoside, malvidin-3-(6ʺ-p-caffeoylglucoside), pelargonidin- 3-(6ʺ-malonylglucoside), peonidin-3-rutinoside-5-glucoside, peonidin-3,5-diglucoside, petunidin-3-rutinoside-5-glucoside. It was found that black wheat has a high concentration of cyanidin-3-glucoside, cyanidin-3,5-di-glucoside, delphinidin- 3-glucoside, delphinidin-3-galactoside, and malvidin-3-glucoside (Sharma N. et al., 2020; Shamanin et al., 2024). Red and white wheat varieties contain cyanidin, delphinidin, malvidin and peonidin anthocyanin derivatives in small concentrations, with the highest content of cyanidin-3-glucoside (Ficco et al., 2014; Garg et al., 2016; Sharma N. et al., 2020). More detailed information on the qualitative and quantitative content of anthocyanin glycosides in grains with different coloration is presented in Table S2

It is assumed that qualitative and quantitative differences in anthocyanin composition may be due to genetic characteristics of the analyzed samples, as well as differences in the equipment used for grain grinding, extraction technologies and quantitative analysis of anthocyanins. Genetic characters cause variation in the qualitative and quantitative composition of anthocyanins in wheat. Each variety has an individual anthocyanin profile (Abdel-Aal, Hucl, 2003). The anthocyanin content is affected by environmental factors like temperature during grain filling period, drought, disease damage (Garg et al., 2022). E.S.M. Abdel-Aal and P. Hucl (2003) studied the anthocyanin content over three crop years. Blue wheat exhibited a reduced effect of environmental factors on anthocyanin content as compared to purple wheat, perhaps due to the location of the anthocyanins in different grain layers. D.V. Bustos et al. (2012) found that anthocyanin content increases rapidly during grain development and decreases before maturity. TAC decreases in the distal position of grains in the spike and when plants are shaded before tillering. On the contrary, TAC increases by halving the spikelet number per spike. Magnesium fertilization and early harvesting increases TAC in purple wheat by 65 and 39 %, respectively. X. Fan et al. (2020) showed that anthocyanin accumulation in purple wheat increases when grown under nitrogen-deficient conditions. According to R. Beleggia et al. (2021), late sowing dates of wheat increases TAC.

## Content of phenolic compounds in colored wheat grain

Phenolic compounds are secondary metabolites that play an important role in the mechanisms of plant defense against UV radiation, pathogen suppression and ensuring the structural integrity of the cell wall. Phenolic acids are the most common class of phenolic compounds (Laddomada et al., 2017), the molecules of which consist of a phenolic ring and a carboxylic acid functional group. There are mainly two groups of phenolic acids in wheat: hydroxybenzoic (vanillic, syringic, p-hydroxybenzoic, gallic, salicylic, protocatechuic, ellagic, and gentisic acid), which have a C6-C1 structure, and hydroxycinnamic acid derivatives (ferulic, cinnamic, coumaric, caffeic, and sinapic acid), which are aromatic compounds with a three-carbon side chain (C6-C3) (Table S3). Individual compounds in each of these groups differ from each other by the presence and structure of side radicals.

Phenolic acids in wheat grains can take the following forms: insoluble, bound by ether and ether-ether bonds to cell wall components such as cellulose, arabinoxylan, lignin, and proteins (about 50–70 % on average); soluble, conjugated to sugars or other low molecular weight components (13–20 %); and soluble, free (0.5–2 %) (Menga et al., 2023).

A lot of studies have shown that the total content of soluble and insoluble phenolic compounds in colored wheat grain increases in the following order: white < purple < blue < black (Kumari et al., 2020; Paznocht et al., 2020) with up to 4–6 times higher content in colored grain compared to uncolored grain (Sharma S. et al., 2018; Kumari et al., 2020; Wang et al., 2020; Garg et al., 2022; Shamanin et al., 2022; Sahu et al., 2023). In general, regardless of the grain color, the grain shells that are removed during milling have the highest content of phenolic acids, as well as anthocyanins and only by using whole wheat flour products you can get all the benefits possible. The quantitative content of soluble and insoluble phenolic compounds in wheat grain with different colors is presented in Table S4.

Ferulic acid is the most abundant compound in wheat grain (65.0–94.9 % of all insoluble bound phenolic compounds) (Ma et al., 2021). It has been shown that the quantitative content of individual phenolic acids in free and bound forms can vary widely depending on the genotype. According to D. Ma et al. (2016), purple grain has higher contents of soluble and insoluble phenolic acids including ferulic acid, vanillin and caffeic acid than blue and red grain.

The study of free phenolic acids content in bran fractions showed that purple grain had maximum TPC (636–1,134 μg/g GAE (gallic acid equivalent)), while in blue and black grain the TPC was from 476 to 874 μg/g and from 495 to 590 μg/g, respectively. Gallic acid (29–33 μg/g), ferulic acid (and isoferulic acid) (59–66 μg/g) and salicylic acid (30–65 μg/g) had the highest content in all samples (Zhang et al., 2018). Among the bound phenolic acids, ferulic (from 1,726 μg/g in black grain to 2,620 μg/g in blue grain) and salicylic acids (from 535 μg/g in blue grain to 906.02 μg/g in black grain, respectively) had the highest content. Overall, phenolic acid content in both free and bound forms as well as AOA gradually decreased in the following order: outer bran > coarse bran > shorts.

V.P. Shamanin et al. (2022) showed that the total phenolic compound content ranged from 446 to 708 mg GAE/100 g in red wheat varieties (189–271 and 227–487 mg GAE/100 g free and bound, respectively), 457 and 767 mg GAE/100 g in blue-grain wheat lines (204 and 247 mg GAE/100 g; 253 and 520 mg GAE/100 g), 353–772 mg GAE/100 g in purple-grain wheat lines (164–248 and 190–432 mg GAE/100 g) and 476– 520 mg GAE/100 g (190–218 and 259–323 mg GAE/100 g) in black-grain wheat lines. In the bound fractions of some purplegrain wheat genotypes, as well as in F4 black wheat hybrids, ferulic and sinapic acids had the highest content: 307–582 and 277–619 μg/g (in purple wheat); 257–424 and 272–450 μg/g (in black wheat); in some genotypes, ellagic or protocatechuic acids were predominant (31–89 and 90–157 μg/g). The free fraction was dominated by gallic, protocatechuic, and – in a number of purple-grain wheat samples – ellagic acid.

According to M. Bueno-Herrera and S. Pérez-Magariño (2020), vanillic (20.3–34.2 μg/g) and trans-ferulic (8.4– 20.2 μg/g) prevailed in the free fraction, while cis- and transferulic (245.1–304.6 μg/g), p-coumaric (8.8–9.9 μg/g) and vanillic acids (6.5–7.2 μg/g) were predominant in the bound fraction. A higher content of phenolic compounds was characteristic of the fine bran fraction (with a particle diameter of 200–800 μm) than in the coarse bran (with a particle diameter of 800–2,000 μm) and flour fractions. Ö.G. Geyik et al. (2023) showed that blue wheat had higher ferulic acid content in the bran fraction than purple and red wheat (2,264, 1,945 and 988 μg/100g, respectively). In the free fractions, p-coumaric acid (11.5 μg/100 g) had the highest content in red wheat and ellagic acid (14.7 and 11.5 μg/100 g, respectively) in purple wheat and black wheat.

D. Ma et al. (2016) studied the accumulation of phenolic acids in white, red, and purple wheat grains. They concluded that the maximum accumulation of ferulic and syringic acids was observed 14 days after flowering, while the levels of p-coumaric and caffeic acids reached the maximum level 7 days after flowering, and the levels of vanillic acid increased gradually during grain filling and reached the maximum level at the ripening stage (35 days after flowering). White wheat had higher phenolic acid contents and relatively high phenolic acid biosynthesis pathway genes (TaPAL1, TaPAL2, TaC3H1, TaC3H2, TaC4H, Ta4CL1, Ta4CL2, TaCOMT1 and TaCOMT2) expression at the early stage, while purple wheat had the highest phenolic acid content and gene expression levels at later stages.

## Antioxidant activity of colored wheat grains

Antioxidants are known to have the ability to neutralize and destroy free radicals that cause damage to cellular structures. The AOA of wheat is caused by anthocyanins, phenolic acids, flavones and flavonols. In vitro and in vivo grain AOA is assessed using a number of methods. The DPPH method is based on the registration of DPPH (2,2-diphenyl-1-picrylhydrazyl) radical reduction upon interaction with antioxidants. Other methods are also used, such as ABTS (decrease in the intensity of absorption by cations of the ABTS radical (2,2′-azino-bis-(3-ethylbenzothiazoline-6-sulfonic acid)), ORAC (oxygen radicals absorbance capacity) – ability to intercept peroxyl radicals, FRAP (ferric reducing antioxidant power) – reduction of trivalent iron complex ion (TPTZ (2, 4,6-3(2-pyridyl)-1,3,5-triazine)) concentration, CUPRAC (cupric reducing antioxidant capacity) – change in optical density in the reduction reaction of Cu2+ to Cu+, PCL – chemiluminescence registration (Ma et al., 2016; Abdel-Aal et al., 2018; Sharma S. et al., 2018; Shamanin et al., 2024). The AOA of colored wheat grain compared to uncolored grain is mainly due to its higher anthocyanin content. C. Hu et al. (2007) found that 69 % of the total free radical scavenging capacity of blue wheat is determined by the content of anthocyanins, while 19 % is attributed to phenolic acids, and the contribution of bound ones is much higher than free ones (Zhang et al., 2018; Shamanin et al., 2022). Cyanidin-3-glucoside has the strongest AOA among anthocyanins – 3.5 times stronger than Trolox (vitamin E analog).

Several studies have shown that blue, purple and black wheat have higher AOA values compared to red and white grain wheat (Ficco et al., 2014; Ma et al., 2016; Sharma S. et al., 2018; Kumari et al., 2020; Wang et al., 2020). The highest AOA values (ABTS and DPPH) are characteristic of black wheat, then decrease in the following order: blue > purple > white, as well as TAC and TPC (Kumari et al., 2020; Sharma A. et al., 2023). AOA values of grains with different coloring determined using different methods are given in Table S5. Since compounds with antioxidant properties are found predominantly in the bran fraction, the AOA of bran is significantly higher than that of whole grain (Siebenhandl et al., 2007; Abdel-Aal et al., 2018; Iannucci et al., 2022; Saini et al., 2023). Y. Jiang et al. (2024) showed that the AOA of superfine flour was 1.18 and 1.62 times higher than that of coarse flour (ABTS and ORAC). A positive correlation was shown between the TPC in flour and AOA (r = 0.769 (ABTS) and r = 0.984 (FRAP)) (Li Y. et al., 2015), between TPC and ABTS (r = 0.97) (Ficco et al., 2014), between soluble phenolic compounds and DPPH (r = 0.65) (Sharma S. et al., 2018). Significant positive correlations were also observed between TAC and AOA (PCL) (r = 0.9) (Sharma S. et al., 2018), individual anthocyanins and ABTS (r = 0.65–0.91) (Shamanin et al., 2024).

## Breeding achievements in Russia in obtaining colored-grain wheat varieties

In Russia, several research institutions are actively working on obtaining promising colored-grain wheat breeding lines and varieties. To date, three purple-grain wheat varieties have passed competitive variety testing and have been included in the register of breeding achievements: Nadira (FRC Kazan Scientific Center of RAS, 2022), Pamyati Konovalov (FSC of Legumes and Groat Crops and Russian State Agrarian University – Moscow Timiryazev Agricultural Academy, 2023), and EF 22 (Omsk State Agrarian University named after P.A. Stolypin, 2024). These varieties are characterized by high levels of TAC and AOA, and therefore can be used for the production of functional foods. The variety Nadira was obtained by individual selection from the hybrid population F3 L.22-95 / Kommissar (Vasilova et al., 2021). L.22-95, which was obtained at the Siberian Research Institute of Agriculture, was a donor of purple grain color. Nadira is recommended for cultivation in the Volga-Vyatka, Middle Volga and Ural regions. It is a medium-maturing variety. The average yield in the competitive variety trial for 2016–2018 was 4.8 t/ha (the standard Yoldyz yield was 4.7 t/ha). The variety is resistant to loose smut, moderately susceptible to leaf rust and powdery mildew. Drought tolerance of the Nadira variety is at the level of standard varieties. The variety contains 13.8 % protein in grain, 25.5 % crude gluten and has baking qualities corresponding to valuable varieties.

The variety Pamyati Konovalov was obtained by individual selection from a hybrid population: (Laval 19 × Granny) × Granny. Laval 19 (Canada) is the donor of purple color. This medium-maturing variety is recommended for cultivation in the Moscow region. Grain yield per plot in 2020–2021 was 451 and 284 g/m2 (the standard variety Zlata yield was 593 and 408 g/m2, respectively). Baking qualities of the variety are satisfactory and good, it is a good filler. The variety is resistant to lodging, septoriosis and fusariosis, moderately resistant to leaf rust (Rubets et al., 2022).

The variety Ivolga fioletovaya (Russian State Agrarian University – Moscow Timiryazev Agricultural Academy) is an isogenic purple wheat line of the variety Ivolga. Grain yield per plot in 2020–2021 was 417 and 358 g/m2, which is lower than that of the Zlata variety. Ivolga fioletovaya is a medium-maturing variety, resistant to lodging, leaf rust and powdery mildew, but susceptible to fusarium and septoriosis (Rubets et al., 2022).

The efforts of scientists of OmSAU and ICIG SB RAS resulted in a number of promising lines from crossing lines of the S29 variety with Ba and Pp genes with Siberian varieties such as Element 22 (with Zn content more than 50 mg/ kg), Aina, Tobolskaya and line BW49880 (CIMMYT, high Zn content) (Gordeeva et al., 2020; Shamanin et al., 2022). These lines are characterized by high TAC (maximum values of 254 and 326 μg/100 g in F4 purple- and black-grain hybrids obtained based on BW 49880), phenolic compounds (767 and 599–772 mg GAE/100 g; 520 and 427–566 mg GAE/100 g total and bound phenolic acids in the blue-grain Blue 10 line (s:C29 4Th(4D)/Element 22) and in purple-grain BC1F4– BC1F5 hybrids of the Element 22 variety, respectively), AOA (CUPRAC) (482–494 mg TE/100 g (trolox equivalent) in the BC1F8 purple-grain lines of the Tobolskaya variety and F4 hybrids of Element 22 and BW 49880), as well as Zn content from 44.5 to 56.5 mg/kg and Fe content from 53.5 to 65.5 mg/ kg (Shamanin et al, 2022, 2024).

The EF 22 variety was created by marker-assisted selection using SSR markers flanking the Pp-D1 and Pp3 genes (Gordeeva et al., 2020) over six years. The donor of purple grain was the i:S29PF line (introgression fragments from Purple Feed) (Arbuzova et al., 1998). EF 22 is a valuable mediumlate variety, which has been included in the State Register for the Ural and West Siberian regions. The average yield for 2016–2020 was 3.12 t/ha, while the Element 22 variety had an average yield of 3.89 t/ha (Pototskaya et al., 2022). A study of the bread characteristics from whole wheat flour of EF 22, breeding lines Blue 10 and Purple 8 (Element 22*2/i:C29PF) showed higher content of phenolic compounds and AOA and lower glycemic index compared to white wheat bread (Koksel et al., 2023).

## Conclusion

The presented review summarized information on the genetic control of the regulation of anthocyanin accumulation and biosynthesis in the pericarp and aleurone layer by the Ba, Pp-1 and Pp3 genes. Information on anthocyanin content, phenolic compounds and AOA levels in wheat with different grain coloration is presented. Purple, blue and black wheat has higher TAC, TPC and AOA than uncolored wheat, and TAC, soluble and insoluble phenolic compounds and AOA values increase in the following order: purple > blue > black wheat. Purple-grain wheat as well as black-grain wheat have a more diverse anthocyanin compositions compared to blue-grain wheat. Colored-grain wheat varieties obtained by Russian breeders are a source of bioactive compounds that play an important role in disease prevention and can serve as a basis for the development of the anthocyanin-biofortified food industry in the internal market and increase the value of exported grain products.

## Conflict of interest

The authors declare no conflict of interest.
